# Women empowerment and uptake of antenatal care services : A meta-analysis of Demographic and Health Surveys from 33 Sub-Saharan African countries

**DOI:** 10.1186/s13690-021-00604-5

**Published:** 2021-05-27

**Authors:** Gebretsadik Shibre, Betregiorigis Zegeye, Helena Yeboah, Ghose Bisjawit, Edward Kwabena Ameyaw, Sanni Yaya

**Affiliations:** 1 Department of Reproductive, Family and Population Health, School of Public Health, Addis Ababa University, Addis Ababa, Ethiopia; 2 HaSET Maternal and Child Health Research Program, Shewa Robit, Ethiopia; 3 School of International Development and Global Studies, University of Ottawa, Ottawa, Canada; 4 The Australian Centre for Public and Population Health Research, Faculty of Health, University of Technology Sydney, Sydney, NSW Australia; 5 The George Institute for Global Health, Imperial College London, London, United Kingdom

**Keywords:** Women’s empowerment, Antenatal care, Global Health, Sub-Saharan Africa, Demographic and Health Surveys

## Abstract

**Background:**

Women empowerment has been linked to increased skilled antenatal care (ANC) service use. However, there is no evidence on the net effect of women empowerment on ANC in the Sub-saharan African (SSA) region. We aim to address the knowledge gap on whether or not women empowerment positively influences the uptake of ANC at the SSA regional level.

**Methods:**

We analyzed the Demographic Health Survey (DHS) datasets from 33 SSA countries. Following the DHS data analysis guideline, we measured women empowerment using two indicators. The first indicator is an index, which comprises decision-making on women’s own health, household purchase and visit to family or relatives whilst disagreeing statements that husband is justified in beating his wife constitutes the second indictor. We performed confounder-adjusted logistic regression analysis for the two indicators with ANC attendance in each of the 33 countries. Then, we pooled the adjusted Odds Ratios (OR) using the random effect model through the two-stage Individual Participant Data meta-analysis technique. Summary findings are reported in OR and corresponding 95 %CI and are presented in a forest plot.

**Results:**

Moderately empowered women had marginally higher odd of skilled ANC service across the SSA region (aOR = 1.19; 95 %CI: 1.03, 1.38, with a prediction interval of 0.58, 2.45). Conversely, being involved in the three decisions (aOR = 1.15; 95 %CI: 0.99, 1.33, with prediction interval 0.57, 2.31), and attitude towards wife-beating (aOR = 0.97; 95 %CI: 0.88, 1.06, with prediction interval of 0.63, 1.48) had no statistically significant relationship with ANC.

**Conclusions:**

Women empowerment did not predict the use of skilled ANC in the context of the SSA region. We recommend that further studies be conducted in order to understand how women empowerment affects skilled ANC service utilization in the region.

## Background

Maternal mortality remains a global health problem and one of the key health challenges in most low and middle-income countries, particularly, sub-Saharan African (SSA) countries. Nine out of the top ten countries that had the highest global estimates of maternal mortality in 2017 were found in the SSA region [1]. Further estimates show that roughly two-third (196,000) of the global maternal deaths were concentrated in Sub-Saharan Africa [2]. Reducing maternal mortality has become one of the key indicators in the measurement of the development status of countries and has been an important aspect of the Sustainable Development Goals (SDGs) as target 1 of the third SDG seeks to reduce the global maternal mortality ratio to less than 70 per 100,000 live births by 2030 [3]. Governments of African states and international organizations, including the African Union (AU), the World Health Organization (WHO), the United Nations Population Fund (UNFPA) and the International Development Research Center (IDRC) inter alia have made concerted effort to reduce maternal deaths by promoting and improving health equity. In spite of all these, there has been limited progress in reducing maternal mortality rates in many countries in SSA [4, 5]. For instance, women in SSA face 1 in 38 chance of dying from pregnancy and childbirth-related causes in comparison to 1 in 5,400 in developed countries [6].

Pregnancy and childbirth-related deaths often occur while a woman is pregnant while giving birth or within 42 days of termination of pregnancy [7]. Almost all these deaths are preventable. Hence, the World Health Organization (WHO) in 2016 recommended that for women to have positive pregnancy experience, there should be a minimum of eight antenatal care (ANC) contacts: one contact in the first trimester, two contacts in the second semester and five contacts in the third trimester [8]. Comprehensive reproductive health care which encompasses antenatal care, skilled delivery and obstetric care play central roles in reducing the maternal mortality rate in various countries and communities. However, many women tend to seek the services of traditional birth attendants (TBAs) or relatives more than they utilize health facilities, resulting in high maternal deaths [9–11].

Women’s access to health care services, including ANC, may depend on either supply and/or demand side factors [12]. The demand side factors are those that relate to the woman’s ability to use maternal health services including indirect transportation costs, health information education, income, household expectations and cultural preferences. Supply side factors are those that influence the provision of services, like price and quality drugs, technology and efficiency of staff. Quantity rationing, long waiting time for service and high cost of service can occur as a result of the interaction of the demand and supply side factors [12, 13]. Most policies and research that tackle health service utilization focus on supply side factors rather than those related to the demand side. However, certain cultural practices and beliefs, low socio-economic status of women and the presence of patriarchal society which reinforces female dependency and male control are known to suppress the utilization of ANC and other healthcare services [9, 14–16].

Evidence shows that as women’s level of empowerment increase in the household, the higher their tendency of utilizing ANC services and delivering with skilled birth attendants (SBAs) [10]. Women’s empowerment is regarded as women’s ability to make effective choices that lead to desired outcomes, and several studies have examined different dimensions of empowerment (using different indicators) and their relationship with development, skilled birth attendance, and maternal and child health (MCH) utilization [17–20]. However, due to the multidimensional nature of empowerment regarding its features and measures, the relationship between empowerment and ANC and other healthcare services as investigated by various scholars have resulted in either positive, negative or mixed outcomes [21, 22].

One study revealed that in five Association of Southeast Asian Nations (ASEAN), women empowerment indicators such as labor force participation, women’s knowledge level and disagreement with reasons to justify wife-beating showed positive relationship with early ANC visits [23]. Similarly, decision-making power as an indicator of empowerment had a negative relationship with early ANC visit in the Philippines [23]. High levels of women’s autonomy failed to enhance the utilization of maternal health services in Nairobi [24]. In a systematic review consisting of 67 studies from developing countries, although findings of the included articles supported the positive relationship between women empowerment and maternal and child health, there was one indicator that showed either significant negative or mixed results [25].

The differences in these empirical findings have been attributed to the lack of a coherent multidimensional conceptual framework for measuring empowerment. Additionally, most studies that show strong positive relationships are conducted in Asia, where cultures which tend to define women’s power over their reproductive health decisions are different from those in the SSA region considering the contextual and cultural dynamics [24, 25]. To this end, the need to explore the nexus between women empowerment and ANC in SSA cannot be overemphasized on account of the high maternal mortality that originates from the sub-region. Using Demographic and Health Survey (DHS) datasets, this study examines the net impact of women’s empowerment on the utilization of ANC services in 33 SSA countries.

## Methods

### Data

We used DHS datasets from 33 SSA countries conducted between 2005 and2018: Angola (2015), Burkina Faso (2010), Benin (2018), Burundi (2017), Congo (2012), DR Congo (2014), Cote d’Ivoire (2012), Cameroon (2010), Gabon (2012), Ghana (2014), Gambia (2013), Guinea (2018), Kenya (2014), Comoros (2012), Liberia (2013), Lesotho (2014), Madagascar (2008), Mali (2018), Mozambique (2011), Niger (2012), Namibia (2013), Rwanda (2015), Sierra Leone (2013), Senegal (2017), Swaziland (2005), Chad (2015), Togo (2013), Uganda (2016), Zimbabwe (2015), Sao Tome e Principe (2009), Ethiopia (2016), Zambia (2016), and South Africa (2016). We selected most recent datasets for each country. We excluded datasets of the Sudan, Malawi, Tanzania, Central African Republic since their datasets do not contain either the response variable or predictor variables or both.

DHS is a nationally representative household survey conducted at regular time intervals to provide countries with updated information on different health topics such as maternal and child health, reproductive health, fertility, nutrition, mortality, and HIV/AIDS to mention just a few. The United States Agency for International Development (USAID) and each country’s statistical agency conduct the survey with technical support from Inner City Fund (ICF) international.

Methods of DHS have been described in detail in the final DHS report of each country and readers can read “appendix” section of the respective countries’ DHS report to learn the methodology. Concisely, it follows a two-stage stratified cluster sampling design. Broad geographic areas like enumeration area (EA) are selected in the first stage through Probability Proportional to Size (PPS) approach, where relatively larger EA is more likely to be selected than smaller EA. In the second stage, a pre-calculated (28 to 30) number of households is selected from each EA. In the selected household, eligible participants (women aged 15 to 49 years and men 15 to 59 years) are interviewed on wide range of areas using questionnaires that are comparable across countries. To capture some useful information on health topics that would be relevant to specific countries, country-specific questions are included in the model questionnaire.

### Variables

The independent variable for the study is women empowerment. Following the DHS data analysis guideline [26], we measured women empowerment using two indicators: decision making on three specific reasons and disagreement with five reasons for justifying wife-beating. For the first indicator, the woman is asked to report whether she participates in decision making regarding her own health care (*person who usually makes decisions on health care for yourself?)*, large household purchase (*person who usually makes decisions on making major household purchases*?), and visits to family or relatives (*person who usually makes decisions on visits to your family or relatives*?). Each of the reasons is dichotomized into 1 and 0, where 1 means that the woman participates in decision making on that particular reason alone, or jointly with her husband, and 0 means she does not participate in the decision making. Finally, a three-category variable was formed out of the three binary variables with values 0 (no empowerment), 1–2 (moderate empowerment) and 3 (high empowerment). Prior evidence followed the same method of creating this variable [27].

To measure whether a woman disagrees to reasons that justify a husband beating his wife, women are asked the following reasons: (a) burning food, (b) arguing with him, (c) going out without telling him, (d) neglecting the children and (e) refusing to have sexual intercourse with him. If the woman disagrees with all of these reasons, they are assumed to be empowered. Therefore, an overall binary variable was created with a value of 1 and 0, where 1 indicates that the woman disagrees to all of the reasons, and 0 indicates she disagrees with 4 or fewer reasons. We computed both indicators of women empowerment for the currently married women which varies by the countries included in the analysis.

To ascertain the relationship between women empowerment and ANC service net of other variables, we accounted for potential confounding factors in our model. A confounding variable is an extraneous factor that interferes with both the already established cause (in our case, women empowerment) and the response variable (in this case, ANC). We included the following variables as confounders: age in years (15-19, 20-29, 30-39, 40-49), education (none, primary, secondary, higher), wealth (poorest, poorer, middle, richer and richest), employment for cash (employment for cash vs. not), regular media exposure (exposed to one or more of the following media at least once a week: reading newspaper or magazine, listening radio, and watching TV vs. not) as well as residence (rural vs. urban). Choice of the confounding was informed by available literature [27, 28].

### Statistical analysis

First, we performed confounder adjusted logistic regression analysis separately for each of the 33 DHS data to produce adjusted Odds Ratio (OR) for the women empowerment-ANC service association. Our regression analysis was strictly accounted for by the three design elements (weight, cluster and strata) to produce an unbiased estimate that is representative of the respective countries. We used the weight, cluster and strata variables already available in the DHSs. We used the ‘svyset’ and ‘svy’ commands in STATA to account for them. We then conducted Individual Participant Data (IPD) meta-analysis following procedure outlined by Burke et al. [29] to determine the pooled effect of women empowerment on skilled ANC service. The IPD meta-analysis has increasingly become an important alternative to the traditional Aggregate Data (AD) meta-analysis. This method of analysis offers researchers greater control on the quality of the data to be analyzed thereby boosting quality of evidence produced. Moreover, meta-analysis done through this approach could not suffer publication bias, one of the most devastating drawbacks of the traditional AD meta-analysis. Finally, the IPD meta-analysis is cost-effective option when analysis through a multi-level model is impossible [29].

We pooled the country specific Odds Ratios (OR) using random effect model to make the findings generalizable. Also, since there exist substantial differences between country variation in terms of context, we chose random effect model over the fixed effect alternative. This is because random effect model allows researchers to assume that the true effect sizes (ORs in this study) vary randomly across studies in the analysis. The inverse-variance approach was used to estimate weights for each study. Unlike in fixed effect model, the random effect model takes the between–study variability into account in the estimation of weights for each individual study. The between–study variability was estimated through the non-iterative, non‐parametric methods of moments (MoM) estimator of DerSimonian and Laird [30]. We reported estimates of OR, the corresponding 95 % confidence interval and predictive interval. The predictive interval measures the extent of uncertainity of similar future studies on the relationship between women empowerment and receipt of skilled antenatal care in SSA. If the predictive intervals include one, this implies that women empowerment will not have effect on receipt of skilled ANC in studies that will be done in the future. We used STATA v 14 for analysis. The *metan stata module* was used for the meta-analysis.

We organized our findings based on the Preferred Reporting of Items for Systematic Review and Meta-analysis for Individual Participant Data (PRISMA-IPD) guideline [31].

### Ethical consideration

Ethical permissions are not required for this study since we used DHS datasets already publicly available. Ethical procedures were the responsibility of the institutions that commissioned, funded, or managed the surveys. All DHS surveys are approved by ICF International as well as an Institutional Review Board (IRB) in respective countries to ensure that the protocols are in compliance with the U.S. Department of Health and Human Services regulations for the protection of human subjects.

## Results

**Fig. 1:**
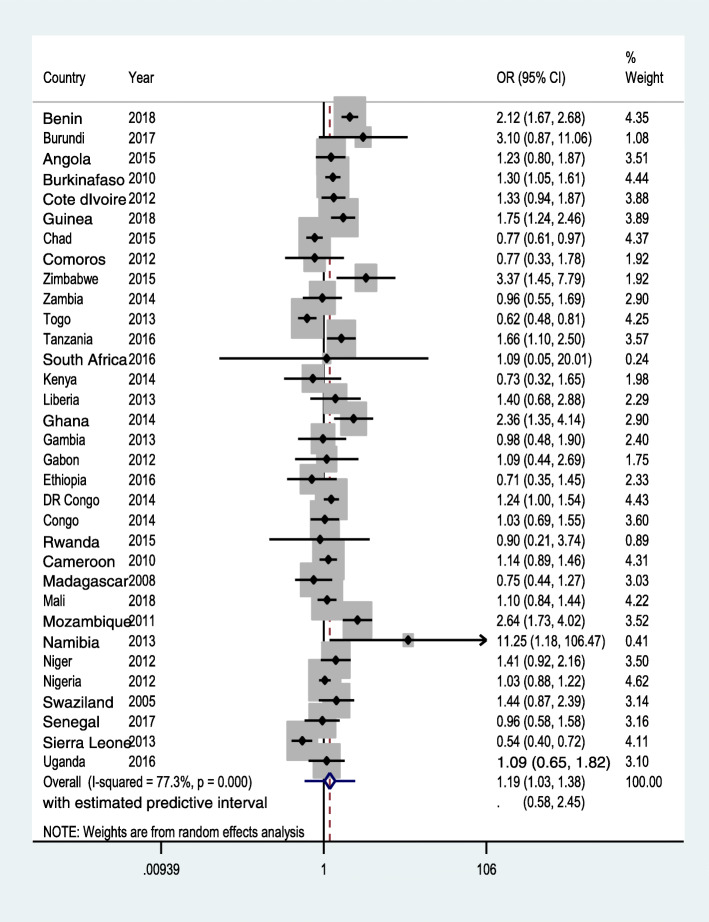
Forest plot showing random-effect meta-analysisbetween moderate women empowerment and ANC, random effectmeta-analysis, SSA, 2021

**Fig. 2:**
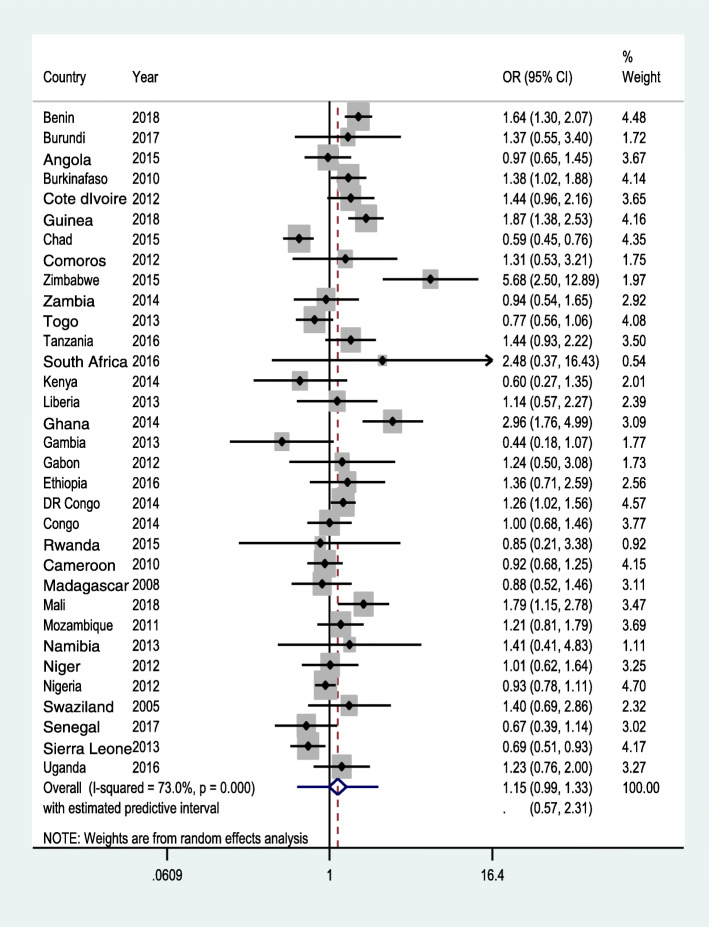
Forest plot showing random-effect meta-analysisbetween high women empowerment and ANC, random effectmeta-analysis, SSA, 2021

**Fig. 3:**
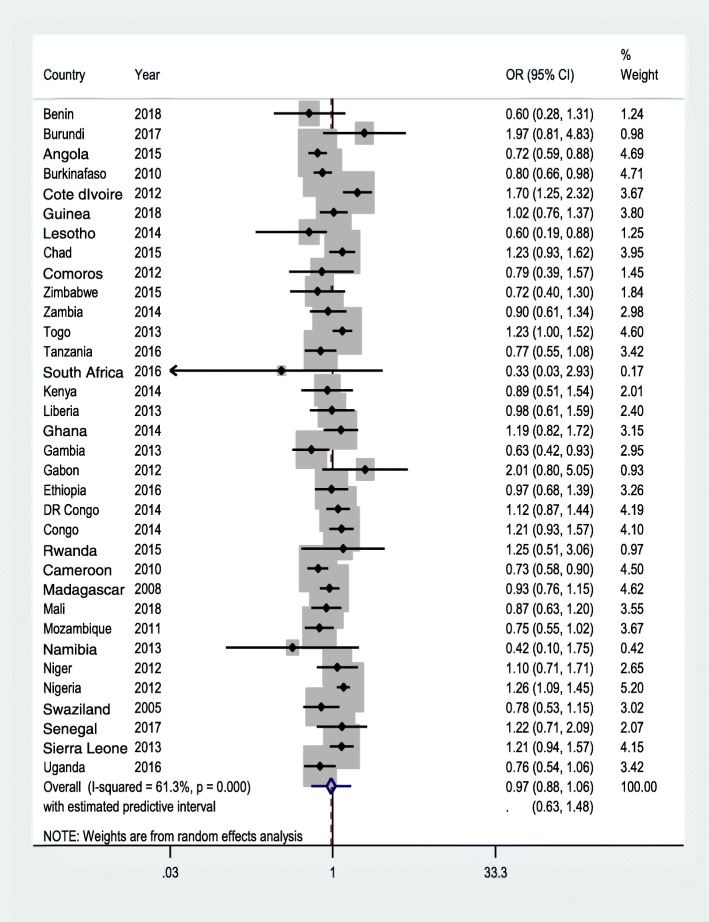
Forest plot showing a random-effect meta-analysis onthe association between wife beating attitude and ANC, random effectmeta-analysis, SSA, 2021

Out of the 33 countries analyzed, moderate women empowerment was associated with higher odd of skilled ANC in nine countries (Benin, Burkina Faso, Guinea, Zimbabwe, Tanzania, Ghana, DR Congo, Mozambique, and Namibia). In three countries, however, (namely Chad, Togo and Sierra Leone), it was associated with lower odd of ANC. In Togo and Cote d’Ivoire, women who disagree to wife-beating justified questions had an increased odd of using skilled ANC (Fig. 3). In the meta-analysis, compared with women who are categorised as not empowered, women who are in the category of moderate empowerment had a higher odd of attending ANC though the prediction interval crossed the null (Fig. 1). However, there was no statistically significant difference in the receipt of skilled ANC between women in the high empowerment group and women categorized as not empowered (Fig. 2). There was also no statistically significant difference between women who disagreed with all five statements of justifying husband’s beating of his wife and women who did not (Fig. 3).

## Discussion

In this study, we attempted to showcase the prediction power of women empowerment on uptake of skilled ANC service in the SSA region. We found no statistically significant difference in terms of uptake of skilled ANC between those who are empowered and those who are not. However, existing evidence has shown women empowerment to raise the chance of uptake of skilled ANC service [27]. The possible hypothesis is that women who are empowered are less likely to experience mistreatment during childbirth [32].

Based on our findings from the 33 SSA countries, women’s participation in decision making and having negative attitude towards wife beating could not help women’s access to ANC services in health facilities. The country level findings showed no substantial similarity in the nature of association between indicators of women empowerment and ANC, as the largest number of countries (26 in wife-beating vs. ANC, 25 in high empowerment vs. ANC and 21 in moderate empowerment and ANC relationships) saw no association between them (Figs. 1, 2 and 3). Similarly, the pooled estimate showed no significant association. Had the study been based on the traditional AD meta-analysis, noticeable difference between study heterogeneities would have made it difficult to observe fairly similar findings across various countries/DHSs, and this again could have seriously affected the conclusion.

Our finding is not consistent with that of prior studies [23, 33–35]. The discordance in the findings pertains partly to the fact that there have not been similar pooled studies on the women empowerment-ANC service relationship at the SSA regional level, thereby complicating direct comparison with preceding studies. For instance, a meta-analysis in SSA involving 31 countries showed a weak association between decision making on major household purchases and at least four ANC [30]. However, it is not clear which method of pooled analysis they followed (fixed vs. random), and also did not fully account for the complex nature of the DHS data). The other difference between this and our study is that our response variable is any skilled ANC service for the most recent birth whereas the Chol et al. used at least four skilled ANC services.

The huge dissimilarity in the construction method for women empowerment could affect our effort of direct comparison of our findings with others. Currently, women empowerment is being approximated in different ways in different studies, showing the lack of standard and accepted tool to measure women empowerment. For instance, in the existing literatures, it has been measured using various concepts including abuses (physical and emotional), whether or not contact with wife’s family member is restricted, not trusting wife with money or limiting freedom of movement, labour force participation, attitude towards domestic violence, Gender Equitable Men scale and autonomy [23, 32, 33, 35, 36]. In this present article, on the other hand, we followed the DHS data analysis manual to create women empowerment, and the proxy indicators in our study are different from that of most other studies [23, 33, 36]. Since all authors do not follow a single guideline to measure women empowerment, it is likely that researchers could come up with different versions of women empowerment, and this greatly hampers production of consistent evidence that could be used for policy making.

Whereas our study could serve as an entry point for furthering regional level effect of women’s empowerment on utilizing skilled ANC, we strongly believe that further studies are required on this line of topic before concluding on the non-influence of women empowerment on uptake of skilled ANC service in SSA. We also recommend the development of a standardized method by which women empowerment can be measured to enable consistency for all researchers who seek to study women empowerment and any other outcome variable of interest. This will further help with the replication and comparison of findings in different context and time.

### Strengths and limitations

The strengths of the study are that, the adoption of the IPD meta-analysis approach for the study elevated the quality of our evidence as it offers us the opportunity to follow fairly similar strategies of analysis in each of the individual countries, and this could help substantially reduce between-study variations in the effect size we measured. However, we used the random effect model so that between studies variation could not be a problem. The approach also granted us sufficient control over the data, something that the traditional meta-analysis technique does not offer. The limitations are that, some DHS datasets were excluded from analysis since they did not contain important variables of interest. The exclusion of these datasets might have affected the conclusion drawn in this article. However, the countries excluded were limited. Finally, we pooled DHSs that were conducted at different time periods, and the effect of women empowerment on ANC might have changed over time. However, the country specific associations of the two variables do not support the effect of time.

## Conclusions

Women empowerment was not associated with the use of skilled ANC in the context of SSA region even though women who were moderately empowered had a higher odd of attending ANC compared with women who were not empowered, the corresponding predictive interval crossed the null and confirmed the lack of statistical association between women empowerment and receipt of the skilled ANC in SSA. We recommend that further studies be conducted in order to understand how women empowerment affects skilled ANC service utilization across the specific countries in SSA. Finally, we recommend the creation of a standard women empowerment measurement indicator that would be used by all countries. This would greatly facilitate the comparison between studies on the effect that women empowerment could have on maternal health care services such as ANC.

## Data Availability

Dataset used in this article is available in the DHS repository available here: https://dhsprogram.com/data/available-datasets.cfm. Data are accessible after registration on the website.

## Abbreviations

ANC: Antenatal care
DHS: Demographic and Health Survey
WHO: World Health Organization
