# Correction to: Women empowerment and uptake of antenatal care services: A meta-analysis of Demographic and Health Surveys from 33 Sub-Saharan African countries

**DOI:** 10.1186/s13690-021-00629-w

**Published:** 2021-06-18

**Authors:** Gebretsadik Shibre, Betregiorgis Zegeye, Helena Yeboah, Ghose Bisjawit, Edward Kwabena Ameyaw, Sanni Yaya

**Affiliations:** 1 Department of Reproductive, Family and Population Health, School of Public Health, Addis Ababa University, Addis Ababa, Ethiopia; 2 HaSET Maternal and Child Health Research Program, Shewa Robit, Ethiopia; 3 School of International Development and Global Studies, University of Ottawa, Ottawa, Canada; 4 The Australian Centre for Public and Population Health Research, Faculty of Health, University of Technology Sydney, Sydney, NSW Australia; 5 The George Institute for Global Health, Imperial College London, London, UK

**Correction to: Archives of Public Health 79, 87 (2021)**

**https://doi.org/10.1186/s13690-021-00604-5**

Following publication of the original article [1], the authors reported an error in the second author’s given name, which was wrongly spelled as “Betregiorigis”.

The correct spelling “Betregiorgis” has been provided in this Correction.

The original article [1] has been updated.
